# Variable Retort Temperature Profiles (VRTPs) and Retortable Pouches as Tools to Minimize Furan Formation in Thermally Processed Food

**DOI:** 10.3390/foods10092205

**Published:** 2021-09-17

**Authors:** Matías Fardella, Cristian Ramírez, Eduardo Caballero, Elizabeth Sánchez, Marlene Pinto, Helena Núñez, Pedro Valencia, Sergio Almonacid, Ricardo Simpson

**Affiliations:** 1 Departamento de Ingeniería Química y Ambiental, Universidad Técnica Federico Santa María, Avenida España 1680, Valparaíso 2390123, Chile; matiasfardella@gmail.com (M.F.); marlene.pinto@usm.cl (M.P.); helena.nunez@usm.cl (H.N.); pedro.valencia@usm.cl (P.V.); sergio.almonacid@usm.cl (S.A.); ricardo.simpson@usm.cl (R.S.); 2 Centro Regional de Estudios en Alimentos Saludables (CREAS), ANID-GORE Valparaíso Proyecto R17A10001, Avenida Universidad 330, Placilla, Curauma, Valparaíso 2373223, Chile; ecaballero@creas.cl; 3 Centro de Biotecnología Dr. Daniel Alkalay Lowitt, Universidad Técnica Federico Santa María, Avda. España 1680, Valparaíso 2390123, Chile; elizabeth.sanchez@usm.cl

**Keywords:** furan formation, thermally processed foods, microstructure, baby food, variable retort temperature profile

## Abstract

Furan and its derivates are present in a wide range of thermally processed foods and are of significant concern in jarred baby and toddler foods. Furan formation is attributed to chemical reactions between a variety of precursors and a high processing temperature. Also, some kinetic models to represent its formation in different food materials have been studied and could predict the furan formation under simulated operating conditions. Therefore, this review aims to analyze and visualize how thermally processed foods might be improved based on optimal control of processing temperature and package design (e.g., retort pouches) to diminish furan formation and maximize quality retention. Many strategies have been studied and applied to reduce furan levels. However, an interesting approach that has not been explored is the thermal process design based on optimum variable retort temperature profiles (VRTPs) and the use of retortable pouches considering the microstructural changes of food along the process. The target of process optimization would be developed by minimizing the microstructural damage of the food product. It could be possible to reduce the furan level and simultaneously preserve the nutritional value through process optimization.

## 1. Introduction

Thermal food sterilization of low-acid food products at a high constant retort temperature profiles (CRTPs) is a standard method that has been used to achieve long-term shelf stability of packaged foods [1]. The processing conditions are mainly based on the use of high temperatures (120–130 °C) for a long duration (usually over 60 min) to ensure a safe and shelf-stable low-acid product. At the same time, texture, taste, flavor, and nutritional value of the food are significantly affected by the extreme processing conditions (time and temperature) and cause a weakening of and damage to the food microstructure [2,3,4,5]. These changes promote the accessibility of nutrients [6,7,8] but also favor the chemical interaction between different nutrients that are now free and able to react, which form other compounds in the food and some compounds are toxic to human health; these compounds are called thermal process contaminants (TPC) [5,9,10,11].

There is strong evidence that TPC exerts adverse toxicological effects and present potential health risks to humans [12]; the most studied of these toxic compounds are furan, acrylamides and advanced glycation end-products (AGEs). Furan compounds represent a wide class of heterocyclic, low-molecular-weight molecules that are formed as products or intermediates in heat-induced reactions. Since 1995, furan and its derivates have received attention because of their classification as “possibly carcinogenic to humans” by the International Agency for Research on Cancer (IARC) [13]. The Food and Drug Administration (FDA) announced that furan was present in many thermally processed foods, such as fruit and vegetable juices, bakery products, canned and jarred foods (for example, soups, sauces, gravies, pasta, vegetables, fish), including jarred food for young children [9,14,15,16]. One of the main concerns is the commercial baby and toddler foods due to the high susceptibility of this consumer group to the amount of furan present in these foods that puts this group at risk because of the amount of food consumed per day relative to their body weight [17].

Thus, we have identified the problem that the commercial food sterilization process has been developed and optimized from a macroscopic point of view. It considers the food as a whole system where the objective function maximizes quality retention (e.g., color, vitamin, and texture) or minimizes processing time, keeping lethality (F_0_) as a constraint. The exposure of the raw materials to high temperatures for long processing times produces important microstructural changes that increase the loss of nutrients such as nonreducing sugars, amino acids, ascorbic acid, polyunsaturated fatty acids (PUFAs), and carotenoids into the medium, favoring conditions that result in the formation of furans. Therefore, the sterilization of food based on food microstructure changes requires a process that can be controlled according to the changes occurring in the food microstructure. Then, a process performed under variable retort temperature profiles (VRTPs) conditions might be an interesting and better alternative compared to the classic CRTPs process. Variable retort temperature profiles mode is based on the optimal temperature control of the retort, allowing the generation of positive and negative temperature ramps [18,19,20,21,22,23,24,25]. The VRTP has been applied and studied as a tool to improve quality retention, but the paramount factor of the application is related to the reduction in processing time [18,19,21,22,23]. Thus far, no studies have reported that VRTPs and food microstructure evolution through the thermal process.

Therefore, this review aims to analyze and visualize how thermally processed foods might be improved based on optimal control of processing temperature and package design (e.g., retort pouches) to diminish furan formation and maximize quality retention.

## 2. Importance of Furan Presence in Thermal Processed Foods

Within the new opinion published by EFSA, [16], to assess exposure from the European food chain, approximately 17,000 data points were collected across the Member States for inclusion in the assessment (versus approximately 5500 in a 2011 EFSA report). The EFSA report confirmed previous assessments that infants are estimated to be the subpopulation exposed to the highest amount of furan on a bodyweight basis, with up to 70% of the exposure coming from ready-to-eat meals. Although there is no reason to believe that there are other major sources in the diet, it should be noted that furan occurrence and concentration have not been determined in all major food categories.

The classification of the samples as described in the published studies is broadly comparable, separating meat (often including poultry) from vegetables, fish, only vegetables, and fruits only or fruits plus vegetables. Most authors have determined relatively higher amounts of furan in meat/vegetables and vegetables only recipes [17,26], and these findings are mirrored by the EFSA 2011 report [15], which depicts lower amounts of furan in cereal-based, fruit and vegetable, and fruit only recipes.

Concerning the amounts, the mean furan exposures for an infant are 0.99 mg (kg bw)^−1^ day^−1^ (at the upper bound scenario) and high exposure (95th percentile) of 1.82 mg (kg bw)^−1^ day^−1^. In comparison, the elderly population has a mean furan exposure of 0.75 mg (kg bw)^−1^ day^−1^ and a 95th percentile exposure of 1.27 mg (kg bw)^−1^ day^−1^. It is critical to consider that estimates from the US are slightly lower because they were based on quantified analytical results generated from consumed foods [27]. The mean exposure and high exposure determined by international and national authorities are overall very consistent, and the differences are likely attributed to different survey methodologies. According to EFSA (2017), the largest contributor to the dietary exposure of furan in older children (aged 4–6 years) was breakfast cereals (40%), and in small children (>6 months), jarred baby foods were the most important contributor to dietary furan exposure. Furthermore, infants and toddlers tend to be more vulnerable due to a higher food intake relative to body weight (b.w.); thus, several studies have focused on baby foods [28,29,30]. According to European monitoring results from 2004 to 2010 [15], the mean lower-upper bound levels (LB/UB) for furan in foods are 31–32 and 0.2–3.2 µg kg^−1^ for baby foods and infant formula, respectively. However, storage conditions and reheating before consumption may influence the furan concentrations of shelf-stable, vegetable-based foods [11]. According to Anese & Suman [31] furan levels in food have been reported to range from a few μg/kg to 7000 μg/kg. The highest furan concentrations were found in roasted and instant coffee, with a mean value of 4579 μg/kg, and in baby foods and soup, which had a maximum concentration of 215 μg/kg. Ready-to-eat meals for infants and small children are the main contributors to the dietary exposure of infants [16]. According to the FAO/WHO Expert Committee on Food Additives for Furan [32], the lower limit for the benchmark dose for a 10% response (BMDL_10_) was 0.96 mg (kg bw)^−1^ day^−1^. From another perspective, the Norwegian Scientific committee for food safety (VKM) established a more conservative BMDL_10_ of 0.14 mg (kg bw)^−1^ day^−1^ and determined that the main source of furan exposure for 6-, 12- and 24-month-old children is jarred baby food [33]. Additionally, margins of exposure (MoE) found in Germany [34], Brazil [17], Chile [35], and Norway [33] have suggested that furan exposure in babies and infants may be considered as a public health risk according to the EFSA.

Altaki et al. [36], sampled baby food from jars containing meat as the main ingredient; they found that the furan concentrations ranged between 7.9 and 64 μg/kg, with a mean value of 35 μg/kg, but that higher levels of furan were found in fish-based baby foods, with a range of 19–84 µg/kg and a mean concentration of 49 µg/kg. The high furan levels found in fish-based baby foods can be attributed to the oxidation and/or degradation of the highly unsaturated fatty acid components of fish muscle during the thermal treatment. Arisseto et al. [17] reported that the levels of furan in the samples varied from not detectable to 95.5 μg/kg, with the samples containing meat and vegetables (beef, carrot, potato) presenting the highest values (95.5 μg/kg) while samples of fruit-based baby food had lower values of furan (1.7 μg/kg). Thus, the industrial treatment of the product, i.e., the pasteurization of fruit and the sterilization of vegetables, influenced the furan content in these samples [36].

Another variable to consider is that the exposure assessment of furan (and alkylated furans) is hampered by several uncertainties, which will lead to either over-or underestimation of exposure. Most of the occurrence data are for purchased products from the shelf and not prepared for consumption. That is the case for preparing baby foods in jars (heating in open or closed jars, with or without stirring, etc.). These factors strongly depend on consumer behavior, are highly variable, and are not quantifiable [16]. Additionally, losses of furan due to evaporation during final preparations have been investigated in a couple of studies with highly variable results [37,38]. 

To summarize ready-to-eat baby food products, it is important to consider that concentration values of furan and furan derivatives are different depending on the feedstock used to formulate the product, which could affect decision making about which furan derivatives are more important to analyze or quantify. In Table 1, we show the ratios of furan/furan derivatives of different jarred baby foods.

The data presented in Table 1 were obtained from Condurso et al. [30] to calculate the furan/furan derivatives ratios for each product. Hence, it is critical to determine which furan derivative is more important to analyze, in addition to furan. For example, Table 1 allows us to mention that furan derivatives with ratios below 1 are more significant than furan. Additionally, furan derivatives with ratios higher than 5 (less than 20% of furan) are not as significant as other furan derivatives. In that way, it is possible to focus the efforts to quantify the more representative furan derivatives of each jarred baby food to be studied. For example, in jarred baby food based on fruits, the more representative furan derivatives are 2-pentylfuran and furfural; on the other hand, for jarred baby food based on meat and vegetables, the more representative furan derivatives are 2-ethylfuran, 2-pentylfuran, furfural and furfuryl alcohol.

To focus the more significant problems, we present Table 2 with data extracted from Sirot et al. [39]. Table 2 shows the contribution (percentages) to the mean lower bound (LW) and upper bound (UB) exposure to furan of several baby foods for children less than 3 years old. The data presented in Table 2 allow us to understand which are the most significant products for children in different stages. For example, between 1–4 months, the mean furan exposure for babies is determined by cereal-based food, vegetable-based ready to eat meals and, infant formula; conversely, among infants/children 13–36 months, the furan exposure is more influenced by vegetable-based and meat/fish based ready to eat products. Despite this study being carried out with French children, it is important to note that the total infant food (thermally processed) decreases with the age of infant; prior to 12 months, the exposure to furans from infant processed food represents 89.4–97.2%, indicating a good reason to focus the research and studies for this type of products.

## 3. Food Microstructure and Its Contribution to Furan Formation during Thermal Processing

Thermal processing plays an important role in food texture and the bioaccessibility of nutrients due to changes at the microstructural level. It has been reported that the total nutrient content can be decreased due to chemical degradation during the thermal process and/or storage. At the same time, the bioaccessibility can increase, as a result of three aspects: (1) cell wall rupture of the vegetable tissues, (2) matrix-nutrient complex dissociation, and (3) molecular transformation in an active structure form [6,7,40,41].

As Aguilera [42] exposed, the food microstructure is a key aspect that should be considered in the food process design because a great part of nutrients are enclosed into the food matrix [6]. For example, Cilla et al. [41] published a review article that presented some cases in which thermal treatment modified the food microstructure to allow nutrient release, which can be considered as a benefit because of the bioaccessibility of sugars, lipids, amino acids, ascorbic acid, and carotenoids was increased due to cell wall rupture. Zhou et al. [43] studied the effect of thermal processing on the nutritional characteristics of an edible fungus. Their results showed that a cooking process using boiling water (100 °C) increased the total polyphenolic compound and free amino acid content and benefitted the in vitro bioaccessibility in terms of total soluble protein and total soluble sugar for control samples. Lemmens et al. [7] showed that thermal treatment (boiling water for 3 min or boiling water for 25 min) of carrots notably favors the amount of β-carotene that is bioaccessible due to a weakening of the cell wall, especially when the most extended thermal treatment. As was reported, bioaccessibility is related to releasing the compound fraction from the food matrix and thus is available for the gastrointestinal tract [6,41]. Nevertheless, it is clear that after a thermal process, the released active compounds from the food matrix are available not only for gastrointestinal tract absorption but also for other types of interactions. 

Based on the aforementioned, it is possible to think that the thermal process produces a weakening of the structure, allowing the nutrient release into the medium (i.e., furan precursors), exposing all of the compounds to the heating medium, and thereby allowing chemical reactions between them and forming furan and its derivates. Based on an in-depth knowledge of the kinetics of microstructural damage, it is possible to imagine that thermal processing could be optimized for the food microstructure at the specific time at which nutrient loss begins, which is important for furan formation. In this sense, one aspect that should be considered is the use of frozen raw materials. Some important microstructural changes are accelerated during the thermal treatment when the raw material is in a frozen state. These changes can facilitate the migration of precursors into the medium at an early stage of the thermal processing and place the precursors in contact with one another for a longer time [6].

## 4. Furan Formation in Thermally Processed Baby Food

Furan is present in a wide variety of foods, which suggests that there are probably multiple routes for its formation, which has been extensively studied [9,11,34,44]. These routes include the thermal decomposition of ascorbic acid, carbohydrate decomposition during the Maillard reaction, and the thermal oxidation of poly-unsaturated fatty acids and carotenoids [17,31,45,46].

### 4.1. Ascorbic Acid

This compound is considered a major precursor of the furan formed during thermal treatment. For example, Limacher et al. [47] reported that in a model-food-based citric acid solution treated at 121 °C for 25 min, the level of furan formed was dependent on the pH of the solution. A furan level of 58 μmol/mol was obtained at pH 4, while a level of 3.7 μmol/mol was obtained at pH 7. A possible explanation is that at pH 7, furan was mainly formed via dehydroascorbic acid as an intermediate. The results obtained in the model food were compared with those of real foods, such as squeezed orange and carrot juices and pumpkin puree treated at 123 °C for 22 min. The results showed that in the case of orange juice enriched with ascorbic acid (56–58 mg added per 100 g), less furan was obtained at the end of the process. The cases of pumpkin puree and carrot juice were different; the furan content increased, and the relationship between the ascorbic acid content and furan formation was unclear [47]. The presence of an oxidizing agent in the media rich in ascorbic acid was shown to favor the formation of furan at higher temperatures (higher than 100 °C). For example, the presence of ferric chloride (an oxidizing agent) was shown to accelerate the formation of furan from ascorbic acid in the temperature range from 100 to 140 °C, which can be an issue for food enriched with ferric ions or food canned in a metal container [48]. Similar routes of furan formation related to the ascorbic acid content were found in the thermal treatment of tomato paste [44].

In terms of the kinetics of furan formation, Palmers et al. [11] showed that furan formation in spinach puree could be fitted to a zero-order model in the temperature range of 110 to 117 °C. Before this study, there was only one approach, namely, that of [48], who developed an experiment to evaluate the formation of furan from ascorbic acid during the heating process under reducing and oxidizing conditions. Between 100 °C and 140 °C, the furan data were fitted with a first-order model.

### 4.2. Lipid and Carotenoids

The other furan precursors are lipids and carotenoids. For lipids, the work developed by Owczarek-Fendor et al. [49], based on the study of the role of fat oxidation in the generation of furan during the thermal treatment of a model food based on a starchy-emulsion system, showed that oil that reached an unrealistically high level of oxidation could promote furan formation. They also found that the fatty acid composition of the oil can have a remarkable influence on furan formation. Additionally, products of lipid oxidation were shown to procure 2-MeF in presence of amino acids [50], and 4-hydroxy-2-butenal was reported as a key intermediate from polyunsaturated fatty acid (PUFA) degradation undergoing cyclization to dihydro-2-furanol, prior to further decomposition to afford furan by the loss of water [51]. Both monounsaturated linoleic and α-linolenic acids were described as being effective in the formation of furan under thermal conditions [52].

Lipid oxidation is most likely one of the most studied pathways for furan formation using model systems. The intermediate degradation product 4-hydroxy-2-butenal rapidly cyclizes to dihydro-2-furanol, which subsequently furnishes furan after the loss of H_2_O. Mixtures of PUFAs reveal that linolenic acid is an efficient precursor of furan [53], particularly in the presence of transition metals that can accelerate lipid oxidation with the subsequent formation of conjugated dienes.

### 4.3. Carbohydrates and Amino Acids

Limacher et al. [54] studied the formation of furan from sugars and specific amino acids (with a focus on Maillard-type reactions) in a model food system and pumpkin puree under thermal conditions simulating sterilization (121 °C for 25 min). Although the study was carried out in the presence of amino acids, the focus was aimed at evaluating sugars. At pH 7, the results showed that the furan level was in the range of 2–17 μmol/mol and that more furan is produced from pentoses than from hexoses. The presence of amino acids was also found to favor the formation of furan from glucose. However, at pH 4, the furan formation was significantly lower, with furan levels below 1.8 μmol/mol. Comparing the results obtained between the model food with those of the pumpkin puree, only 21% of the total furan formed in pumpkin puree was generated by the sugar-amino acid reaction route, which implies that the remaining 79% was generated from precursors other than sugar. Thermal decomposition of amino acids leads to reactive glycolaldehyde and acetaldehyde as transient intermediates, resulting in furan [55]. In model systems, the levels of 2-MeF obtained by thermal decomposition of pure glucose, fructose, or arabinose were significantly lower than those of furan; however, the levels of 2-MeF were shown to increase to levels that were higher than those of furan with heat treatments of the same sugars in the presence of alanine and serine. Amino acids subjected to oxidation may also procure reactive carbon-2 units such as glycolaldehyde and acetaldehyde. Carbohydrates reacting with amino acids in a classical thermally driven Maillard process furnish 3,4-dihydroxybutenal that subsequently cyclizes to the furanoic backbone [56]. However, amino acids alone (for example, alanine, serine) or glucose alone was shown to represent only a minor source of furan. 

### 4.4. Other Precursors and Intermediates in Furan Formation

An alternative route leading to furan was proposed [57] that involves a Cannizzarro reaction of 2-furfural to afford 2-furoic acid (2-FA). The loss of CO_2_ directly affords furan in thermally treated food. Recently, Delatour et al. [58] carried out the first study showing the role of 2-furoic acid (2-FA) and 2-furfuryl alcohol (2-FOL) in the formation of furan and 2-MeF, respectively, under thermal dry conditions for the temperature range 150–190 °C. Furan is obtained by decarboxylation of 2-FA, while 2-MeF is obtained by dehydration of the lateral chain of 2-FOL. Meanwhile, both 2-FA and 2-FOL did not act as precursors of 3-MeF. 

On the other hand, Adams et al. [50] demonstrated that secondary oxidation products such as α,β-unsaturated aldehydes can furnish 2-MeF via an oxidative, free radical driven mechanism when heated under dry conditions. The intermediate, (Z)-4-hydroxy2-alkenal, cyclizes to furnish the furan moiety that is catalyzed by amino acids or peptides, most likely through the hydrogen bonding ability of amino acids. These studies indicate that in a typical food recipe, the presence of lipids alone will not determine the yield of furan in thermal processing, and the focus needs to remain on the factors that influence lipid oxidation. 

## 5. Kinetic Model Applied to Describe Furan Formation in Food Materials

Given the toxicological properties of furan, actions should be taken to minimize exposure to an acceptable level. Accordingly, knowing the kinetics of furan formation in thermally processed foods is an interesting and powerful tool to adjust process parameters so that furan concentration in the final product can be control or reduce. However, to date, there is very little information on this subject. Below we proceed to describe some models proposed in the literature:

Palmers et al. [11] worked with furan formation in sterilization of spinach puree and described by an empirical a zero-order model. The authors compared the amounts of furan formed in classic sterilization using three temperatures (110, 117 and 124 °C) versus high-pressure high temperature (HPHT) process. The rate of formation was modeled according to the rate law:

(1) $$r=\frac{dF}{dt}=k^{n}$$

where *r* is the rate of furan formation, *F* the furan concentration at time *t*, *k* is the reaction rate constant at the selected pressure and temperature levels, and *n* the order of the equation (0 for this case). The reaction rate constants at each processing temperature were 0.035, 0.071 and 0.142 ng furan/g of pureé/min at 110, 117 and 124 °C, respectively.

The temperature dependence of the reaction rate constant was described by the Arrhenius law:

(2) $$k_{T}=k_{T_{ref}}\exp\left(\frac{-E_{a}}{R}\left(\frac{1}{T}-\frac{1}{T_{ref}}\right)\right)$$

in terms of activation energy *E_a_* (Equation (2)), where *k_T_* and *k_Tref_* represent the reaction rate constant at the selected processing temperature *T* and the reference temperature *k_Tref_*, respectively, and *R* the universal gas constant (8.314 J/K mol). In general, the results showed a good fit of the model with R^2^ adjusted of 0.958.

Mariotti-Celis et al. [59] developed a kinetic study of furan formation in a wheat flour-based model system during frying. The mathematic model used was based on a modification of the Gompertz equation:

(3) $$F=F_{eq}\exp\left(-\exp\left[\frac{k*e}{F_{eq}}\left(\lambda -t\right)+1\right]\right)$$

where *F* is the furan content (ng furan/g dried defatted solids), the parameter *F_eq_* is related to the asymptotic or equilibrium value of the function when *t→∞, k* (ng furan/g defatted solid min) is the maximum rate (determined by the slope of the steepest tangent to the exponential phase), *e* is the Euler number, and *λ* (min) is the lag time (determined at the interception of the base line with the steepest tangent line in the exponential phase).

Palmers et al., [60] focused on the formation of furan on orange and mango juice during storage, applying an empirical logistic model that described the furan concentration:

(4) $$F=\frac{F_{s}}{1+\exp\left(\frac{4*k_{\max}}{F_{s}}\left(\lambda -t\right)+2\right)}$$

where *F* is the furan concentration at storage time *t*, *F_s_* a plateau concentration at long storage times, *k_max_* the maximum reaction rate constant, and *λ* the duration of the lag phase, for a given storage temperature. The temperature dependence of the maximum reaction rate constant *k_max_* followed the Arrhenius equation:

(5) $$k_{\max}=k_{T_{ref}}\exp\left(\frac{-E_{a}}{R}\left(\frac{1}{T}-\frac{1}{T_{ref}}\right)\right)$$

The duration of the lag phase was described as a linear function of the storage temperature:

(6) $$\lambda_{T}=\lambda_{ref}+b_{T}\left(T-T_{ref}\right)$$

where the activation energy *E_a_* and parameter *b_T_* represent a quantitative measure for the temperature-dependencies of the maximum reaction rate constant *k_max_* and the lag time *k*, respectively.

Mogol & Gökmen [48] modeled the rate of furan formation constant by analyzing the differential equations according to a possible network pathway proposed by Perez-Locas & Yaylayan, [58]. Degradation of ascorbic acid (AA) and dehydroascorbic acid (DHAA) was observed under low moisture conditions, elevated temperatures (100, 120 & 140 °C) and oxidizing or reducing conditions. Kinetic constants, estimated by multi-response modeling, stated that adding Fe (oxidizing agent) significantly increased furan formation rate constant, namely 369-fold higher than that of the control model at 100 °C. Degradation of AA and DHAA leads to the formation of diketogluconic acid (DKG) and an intermediate. These two molecules also react to form furan [*F*].

(7) $$\frac{d\left[F\right]}{dt}=k_{7}\left[DKG\right]-k_{8}\left[F\right]$$

The different rate constants (*k*_7_) at 100 °C, 120 °C and, 140 °C were 2.13 × 10^−5^ ± 8.71 × 10^−6^, 5.58 × 10^−3^ ± 7.56 × 10^−4^ min^−1^ and 4.22 × 10^−2^ ± 2.60 × 10^−2^ min^−1^, respectively. 

Gül Akillioğlu et al. [44] investigated the kinetic behavior of furan formation based on the mechanism reaction network for ascorbic acid (as a precursor) proposed by [55] in tomato pulp during heating at 70 °C, 80 °C and 90 °C. 

The differential equations for a simplified reaction mechanism for furan formation considered in this study are presented below

(8) $$\frac{d\left[AA\right]}{dt}=-k_{1}\left[AA\right]+k_{2}\left[DHAA\right]$$

(9) $$\frac{d\left[DHAA\right]}{dt}=-\left(k_{2}+k_{3}+k_{4}\right)\left[DHAA\right]+k_{1}\left[AA\right]$$

(10) $$\frac{d\left[F\right]}{dt}=k_{3}\left[DHAA\right]-k_{5}\left[F\right]$$

where constants *k*_1_, *k*_2_ and, *k*_3_ were obtained by solving the differential equations presented. The temperature dependence of rate constants was also determined, obtaining the activation energy for *AA* degradation (45.13 kJ/mol) and furan formation (40.58 kJ/mol). Interestingly, the results reported here are related to the asymptotical behavior of furan formation along the processing time for the three temperatures studied (70,80 and, 90 °C), which differ from other studies such as [11], where the behavior was linear (order 0).

The importance of counting with reliable models that describe the furan formation according to temperature is related to the possibility of simulation of the sterilization process to predict the furan content. As we can estimate the quality retention of nutrients based on D-value and z-value calculated for nutrients, in the same way, using *E_a_* and *k_ref_* is possible to simulate the evolution of furan inside the can or jar. With this information, it is possible to optimize the VRTP able to minimize the furan formation. 

The kinetic modeling of furan generation should be considered a powerful tool to control its final occurrence in foods, since kinetic parameters can help predict the influence of processing conditions over its concentration. In this sense, knowing the kinetics of furan formation in foods could be considered a useful tool for designing thermal processes that control the level of furan content in the final product, so the process can be more efficient in reducing furan formation.

## 6. Variable Retort Temperature Profiles v/s Constant Retort Temperature Profiles: De-Signing Processes to Delay Precursors Release

The main objective of thermal sterilization technology is to produce safe and high-quality food at a price that consumers are willing to pay. In this way, it has contributed significantly to the nutritional well-being of much of the global population [61]. It is worth noting that temperature must be measured at the slowest-heating zone of the food (also called the cold spot) located typically at the geometrical center of the package [1]. In this sense, thermal food processing is a function of several factors, such as the product heating rate (thermophysical food properties), package size and shape, surface heat transfer coefficient, initial temperature of the food, heating medium, come up time (CUT), retort temperature (operational temperature), and required lethality. As the thermal process inactivates microorganisms, nutrients such as vitamins are similarly affected. The thermolability of nutrients is measured and characterized by the Arrhenius model or, more typically, through z-value (temperature necessary to reduce in one log cycle the D-value) and D-value (time need to reduce in one log cycle the nutrient concentration or microorganism content at a specific temperature) [1,23].

In general, thermal food sterilization is performed under a CRTP. In a CRT process, the typical temperature used is 121.1 °C, and the processing time required to achieve a lethality value F_0_ (F_0_ defined as a thermal treatment that allows the reduction of 12 decimal reduction of *Clostridium botulinum* spores) higher than 3 min will be dependent on the kind of food and the size and shape of the package [1]. F_0_-values lower than 3 min are synonymous with microbiologically unsafe food. However, maintaining the F_0_-value as a constraint, it is possible to modify the retort temperature and thus modify the processing time. These different retorts temperature-processing times are known as equivalent lethality processes or isolethal processes [20].

**Figure 1:**
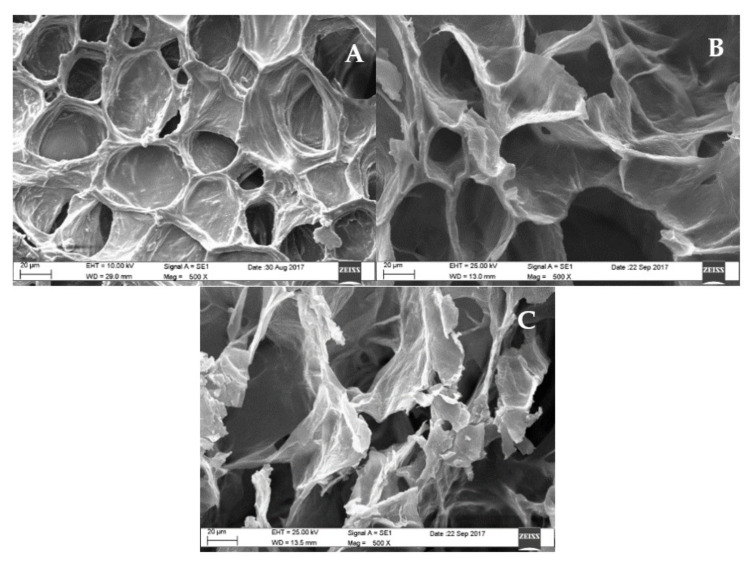
Micrographs of carrots cubes of 1 cm × 1 cm × 1cm: (**A**) raw; (**B**) processed at 110 °C and (**C**) processed at 120 °C. Data source: Hauck et al. [62].

An equivalent lethality curve can be used as a tool to optimize the process in terms of processing time and/or quality retention (surface quality retention or cooking-value, both by knowing the D-value and z-value of the nutrient). However, these processes are not flexible enough in terms of specific control over the change of microstructure. For example, Figure 1 presents micrographs of carrots raw and processed with CRTP at 110 °C and 120 °C keeping *F_0_* = 6 min as a target [62]. The process at 120 °C required a shorter processing time than those performed at 110 °C; due to the high temperature, the structure was significantly damaged. Thus, even when the process at 120 °C was the shortest, the exposure to this temperature favored cell damage. This result could suggest that using lower temperatures during the canning process could reduce the contaminant formation. However, the extended exposure time to the precursor could favor the furan formation. Then, knowledge regarding the moment that the microstructure begins to break could be key for the process redesign because, from that moment, the process temperature could be diminished to avoid the reactions that form furan.

The VRTP is an optimization of the thermal process, in which the temperature inside the retort is modulated during the process [18,19,22,23,24,63]. The application of VRT has been shown to shorten the processing time by 20–30%, implying a 5–15% improvement in the surface quality of canned food compared with CRT processing [64,65]. Almonacid et al., 1993 [19] showed that VRTPs could achieve quality retention, lethality, and energy consumption equivalent to a CRTP with a processing time reduction of 5 to 14 min. Through VRTPs, it is possible to reduce the overprocessing of food and thereby minimize furan formation. Simpson et al. [22] reported some VRTPs that can be perfectly performed in a retort with an adequate control system. In the same work, it was demonstrated that when two products are combined, they cannot be processed using CRTP but can be processed using VRTP without compromising their quality. The VRTP is a powerful tool that can be utilized to reduce processing time, maximize quality retention, and, more importantly, reduce the furan formation in foods to the recommended values. Other authors [25] have shown that applying a VRTP during pasteurization of canned papaya pure was possible to reduce the processing time 33% compared with CRTP; these findings implied that the retention of vitamin C was notably increased from 19% for a CRTP to 46% for a VRTP, which clearly showed an improvement in quality retention applying this optimization.

According to Simpson et al. [23], VRTPs can be more successful in reducing processing time when applied to food with a heating rate (*f_h_-values*) between 20 to 57 min, i.e., foods with higher thermal diffusivities, or foods packaged in small containers. In those cases, the simulations demonstrated that the expected time reduction could attain values of between 27 to 22%. Then, baby food can be considered in the range of *f_h_*-values between 20 to 57 min. Therefore, the application of VRTP can be promising for this kind of product.

**Figure 2:**
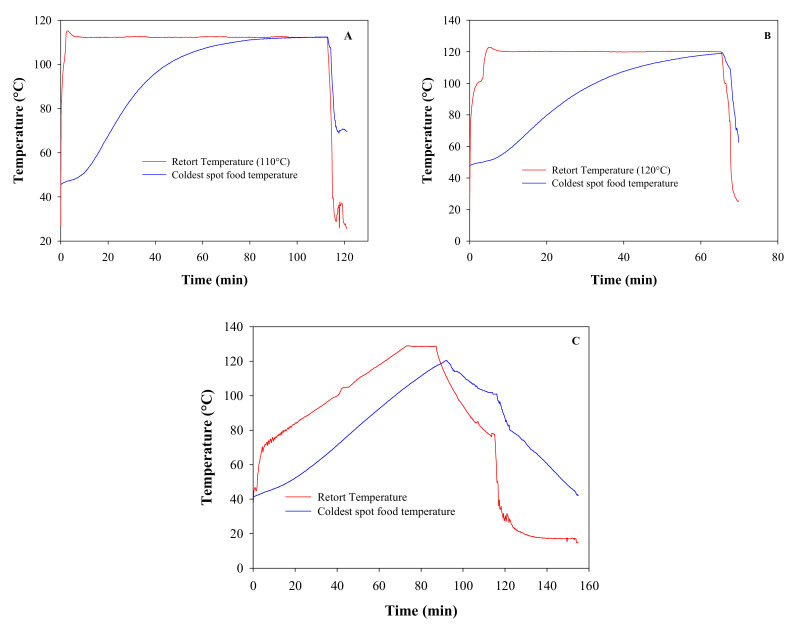
CRT and VRT profiles obtained from thermal sterilization of bentonite with gallic acid as the quality component at (**A**) 110 °C, (**B**) 120 °C and (**C**) VRT. Data source: Salgado et al. [66].

For example, Figure 2 presents three temperature profiles for bentonite with gallic acid as a quality component for processing performed with CRTP at 110 °C, 120 °C, and VRTP [66]. The results showed that the process at 110°C was the longest, attained quality retention of 29% (gallic acid present in the can at the end of the process), while the process at 120 °C was the shortest attained quality retention of 33%. VRTP took an intermediate time to attain quality retention of 35%. These findings show the high potential of VRTP to improve quality (avoid over-processing) and reduce processing time.

## 7. Package Design to Improve Quality Retention in Processed Food

Finally, an interesting factor that can be modified into the thermal food processing is the kind of packaging used for the food. Historically, in the case of baby food, jars have been used as packaging. However, retortable flexible retort pouches have many advantages over jars for the processor, distributor, retailer, and consumer [61,67]. The retortable pouch is a flexible laminated pouch that can withstand thermal processing temperatures and combines the advantages of metal cans and plastic packages [1]. The thin cross-section of retortable pouches allows for rapid heat penetration to the coldest point during thermal processing and, therefore, minimal surface overcooking occurs [68].

For this reason, food products that are packaged in retort pouches require approximately half the cooking time of those packaged in conventional cans [69]. However, adequate pressure control must be provided in the retort to avoid damage to the package [70]. For example, Shah et al. [69] processed a traditional meat product of Kashmir, India, using retortable pouches at 121 °C from *F_0_*-value = 7 to 11 min, and their results showed that all the samples presented good sensory acceptability and were microbiologically safe for 12 months, demonstrating the feasibility of using this kind of packaging in ready to eat foods. Similar results in terms of final quality have been reported by [71,72]. In this sense, it is highly expected that this kind of package allows reducing the furan formation due to the short time that food needs to be exposed to a higher temperature to get the targeted lethality.

## 8. Final Remarks and Conclusions

Furan (C_4_H_4_O) is classified as a possible carcinogen to humans and is present in many foods, including baby/toddler jarred (thermally processed) foods. The latter indicates that babies are the most significant risk group due to the relatively high concentrations of furan consumption and because jarred food is the main part of their daily diet. According to the EFSA opinion and other studies, the MOE for babies is far below the range for safe consumption. Therefore, although food manufacturers and food scientists have attempted to identify and prioritize parameters that may lead to furan formation to implement mitigation measures, the problem is far from being solved. A paramount challenge in establishing clear principles for mitigation is that many different reaction pathways and multiple precursors are involved, for example, lipids, amino acids, and carbohydrates. In this sense, some kinetics models have been implemented to understand the different operation condition and mechanism that promote the furan formation. Another big challenge is to take mitigation strategies to an industrial level and maintain (or at least not increase) the final product price. 

Classic CRTP jarred/canned food sterilization is the processing method that generates the high formation of furans. The overheating of the walls of jars/cans (indispensable to sterilize the coldest point of the product) is a problem that the VRT process can mitigate or at least reduce. In addition, relatively new packages such as retortable pouches are some of the new trends that could help to avoid overheating the package walls. Therefore, it is important to consider the kinetics of the microstructure as a function of temperature for different raw materials of baby food. With this information, it is possible to implement a VRTP optimization process in conjunction with a container design with a high area per unit volume ratio (e.g., retortable pouches), which could significantly reduce the levels of furan formation and simultaneously preserve the nutritional value while maintaining the sensory properties such as odor, color and, taste. Additionally, it could be a valuable instrument to obtain adequate texture modifications of the food microstructure for elderly and aging people according to their aging physical needs and disorders.

## Figures and Tables

**Table 1:** Furan concentration and ratios furan/furan derivatives of different jarred baby food. Data source: Condurso et al. 2018.

Product	Furan Concentration (ng/g of Product)	Ratio Furan/2-Methylfuran	Ratio Furan/2-Ethylfuran	Ratio Furan/2-Butylfuran	Ratio Furan/2-Pentylfuran	Ratio Furan/2-Acetylfuran	Ratio Furan/Furfural	Ratio Furan/Furfuryl Alcohol
Apple-Banana	3.89	12.55	10.81	---	0.79	---	0.48	---
Apple	3.95	13.17	11.29	---	0.81	---	0.49	---
Apple-Banana	3.78	11.81	10.50	---	0.76	---	0.47	---
Apple-Apricot	4.16	13.00	11.24	65.00	0.83	36.17	0.51	3.33
Multifruit	4.12	12.48	11.44	63.38	0.82	34.05	0.50	3.22
Pear	4.02	12.56	10.86	---	0.81	---	0.49	---
Pear	4.19	12.70	11.32	---	0.85	---	0.51	---
Pear	4.03	12.21	11.19	62.97	0.80	---	0.49	---
Veal	30.14	8.56	2.68	20.64	2.15	5.90	1.08	1.47
Veal	29.94	8.60	2.65	19.32	2.16	5.96	1.07	1.47
Veal	31.04	8.65	2.77	20.56	2.26	5.91	1.13	1.45
Veal	24.05	7.99	2.67	18.64	2.35	5.00	0.96	1.42
Beef	28.91	8.33	2.58	19.40	2.09	5.55	1.05	1.37
Beef	30.39	8.61	2.69	19.36	2.18	5.74	1.09	1.44
Beef	29.15	8.38	2.59	18.45	2.14	5.52	1.07	1.43
Beef	23.92	8.00	2.63	18.26	2.31	4.87	0.98	1.50
Chicken	29.45	8.39	2.61	18.64	2.10	5.69	1.06	1.41
Chicken	23.96	7.93	2.61	18.29	2.33	5.00	0.96	1.45
Chicken	19.19	8.57	2.44	19.38	2.44	7.65	0.94	1.95
Chicken	23.54	7.82	2.48	18.39	2.28	4.88	0.95	1.43
Turkey	30.09	8.62	2.66	19.80	2.15	5.80	1.11	1.45
Turkey	24.59	8.12	2.61	19.06	2.39	4.97	0.99	1.49
Turkey	18.53	8.46	2.34	20.36	2.34	7.89	0.91	1.85
Turkey	23.89	7.91	2.52	18.52	2.31	4.92	0.94	1.45

**Table 2:** Contribution of different foods to the LW and UB exposure to furan. Data source: Sirot et al., 2019.

Foods	1–4 Months		5–7 Months		7–12 Months		13–36 Months	
	LB	UB	LB	UB	LB	UB	LB	UB
Milk-based beverage	3.5	2.2	2.4	2.3	1.5	1.4	0.7	0.7
Cereals-based food	22.6	14.4	4.9	4.6	2.9	2.7	1.8	1.6
Milk-based dessert	0.2	0.4	0.4	1.2	0.4	1.3	0.1	0.4
fruit juice	0.9	0.6	0.6	0.6	0.4	0.4	0.2	0.2
Growing-up milk	---	---	---	---	0.2	0.6	0.8	2.4
Soup puree	4.8	3.0	3.1	2.9	3.6	3.4	3.5	3.1
Fruit puree	3.1	2.0	4.8	4.4	3.5	3.3	1.7	1.5
Vegetable-based ready to eat meal	33.2	21.1	38.5	35.8	23.0	21.7	15.4	13.6
Meat/fish based ready to eat meal	---	---	28.9	26.9	52.3	49.4	28.6	25.2
Infant formula	25.9	49.4	0.3	0.8	0.0	0.1	---	---
Follow-on formula	3.0	2.5	10.8	13.8	3.7	5.1	0.2	0.3
Total Infant foods	97.2	95.4	94.7	93.3	91.5	89.4	53.2	48.9
Total common food	2.8	4.6	5.3	6.7	8.5	10.6	46.8	51.2

## References

[1] Holdsworth S.D., Simpson R. (2016). Thermal Processing of Packaged Foods.

[2] Joardder M.U.H., Kumar C., Karim M.A. (2015). Food structure: Its formation and relationships with other properties. Crit. Rev. Food Sci. Nutr..

[3] Karim M.A., Rahman M.M., Pham N.D., Fawzia S. (2018). 3-Food Microstructure as affected by processing and its effect on quality and stability. Woodhead Publishing Series in Food Science, Technology and Nutrition.

[4] Ko W.-C., Liu W.-C., Tsang Y.-T., Hsieh C.-W. (2007). Kinetics of winter mushrooms (*Flammulina velutipes*) microstructure and quality changes during thermal processing. J. Food Eng..

[5] Mariotti M.S., Granby K., Rozowski J., Pedreschi F. (2013). Furan: A critical heat induced dietary contaminant. Food Funct..

[6] Parada J., Aguilera J. (2007). Food Microstructure Affects the Bioavailability of Several Nutrients. J. Food Sci..

[7] Lemmens L., Van Buggenhout S., Van Loey A.M., Hendrickx M.E. (2010). Particle Size Reduction Leading to Cell Wall Rupture Is More Important for the β-Carotene Bioaccessibility of Raw Compared to Thermally Processed Carrots. J. Agric. Food Chem..

[8] Ma Z., Boye J.I., Simpson B.K., Prasher S.O., Monpetit D., Malcolmson L. (2011). Thermal processing effects on the functional properties and microstructure of lentil, chickpea, and pea flours. Food Res. Int..

[9] Crews C., Castle L. (2007). A review of the occurrence, formation and analysis of furan in heat-processed foods. Trends Food Sci. Technol..

[10] Paciulli M., Ganino T., Carini E., Pellegrini N., Pugliese A., Chiavaro E. (2016). Effect of different cooking methods on structure and quality of industrially frozen carrots. J. Food Sci. Technol..

[11] Palmers S., Grauwet T., Celus M., Kebede B.T., Hendrickx M.E., Van Loey A. (2015). Furan formation as a function of pressure, temperature and time conditions in spinach purée. LWT.

[12] Mogol B.A., Gökmen V. (2016). Thermal process contaminants: Acrylamide, chloropropanols and furan. Curr. Opin. Food Sci..

[13] Rychlý R., Nývlt J. (1974). Measuring and Calculating Heat of Crystallisation. Cryst. Res. Technol..

[14] FDA Exploratory Data on Furan in Food: Individual Food Products. https://www.fda.gov/food/chemicals/exploratory-data-furan-food.

[15] (2012). EFSA Scientific Committee Statement on the applicability of the Margin of Exposure approach for the safety assessment of impurities which are both genotoxic and carcinogenic in substances added to food/feed. EFSA J..

[16] Knutsen H.K., Alexander J., Barregård L., Bignami M., Brüschweiler B., Ceccatelli S., Cottrill B., Dinovi M., Edler L., Grasl-Kraupp B. (2017). Risks for public health related to the presence of furan and methylfurans in food. EFSA J..

[17] Arisseto A.P., Vicente E., Toledo M.D.F. (2010). Determination of furan levels in commercial samples of baby food from Brazil and preliminary risk assessment. Food Addit. Contam. Part A.

[18] Chen C., Ramaswamy H. (2002). Modeling and Optimization of Constant Retort Temperature (CRT) Thermal Processing Using Coupled Neural Networks and Genetic Algorithms. J. Food Process. Eng..

[19] Almonacid-Merino S.F., Simpson R., Torres J.A. (1993). Time-Variable Retort Temperature Profiles for Cylindrical Cans: Batch Process Time, Energy Consumption and Quality Retention Model. J. Food Process. Eng..

[20] Simpson R., Abakarov A., Teixeira A. (2008). Variable retort temperature optimization using adaptive random search techniques. Food Control..

[21] Ansorena M., Salvadori V. (2011). Optimization of thermal processing of canned mussels. Food Sci. Technol. Int..

[22] Simpson R., Ramirez C., Jiménez D., Almonacid S., Nuñez H., Angulo A. (2019). Simultaneous multi-product sterilization: Revisited, explored, and optimized. J. Food Eng..

[23] Simpson R., Jiménez D., Almonacid S., Nuñez H., Pinto M., Ramírez C., Vega-Castro O., Fuentes L., Angulo A. (2020). Assessment and outlook of variable retort temperature profiles for the thermal processing of packaged foods: Plant productivity, product quality, and energy consumption. J. Food Eng..

[24] Durance T.D. (1997). Improving canned food quality with variable retort temperature processes. Trends Food Sci. Technol..

[25] Avila-Gaxiola E., Delgado-Vargas F., Zazueta-Niebla J., López-Angulo G., Vega-García M., Caro-Corrales J. (2015). Variable Retort Temperature Profiles for Canned Papaya Puree. J. Food Process. Eng..

[26] Zöller O., Sager F., Reinhard H. (2007). Furan in food: Headspace method and product survey. Food Addit. Contam..

[27] FAO/WHO (2011). Safety Evaluation of Certain Contaminants in Food/Prepared by the Seventy-Second Meeting of the Joint FAO/WHO Expert Committe of Food Aditives (JECFA).

[28] Scholl G., Humblet M.-F., Scippo M.-L., De Pauw E., Eppe G., Saegerman C. (2013). Preliminary assessment of the risk linked to furan ingestion by babies consuming only ready-to-eat food. Food Addit. Contam. Part A.

[29] Pugajeva I., Rozentale I., Viksna A., Bartkiene E., Bartkevics V. (2016). The application of headspace gas chromatography coupled to tandem quadrupole mass spectrometry for the analysis of furan in baby food samples. Food Chem..

[30] Condurso C., Cincotta F., Verzera A. (2018). Determination of furan and furan derivatives in baby food. Food Chem..

[31] Anese M., Suman M. (2013). Mitigation strategies of furan and 5-hydroxymethylfurfural in food. Food Res. Int..

[32] FAO/WHO (2010). FAO/WHO Summary Report of the 72nd Meeting of the Joint FAO/WHO ExpertCommittee on Food Additives (JECFA).

[33] VKM Report (2012). Risk assessment of furan exposure in the Norwegian population. Nor. Sci. Comm. Food Saf..

[34] Lachenmeier D.W., Reusch H., Kuballa T. (2009). Risk assessment of furan in commercially jarred baby foods, including insights into its occurrence and formation in freshly home-cooked foods for infants and young children. Food Addit. Contam. Part A.

[35] Mariotti M.S., Toledo C., Hevia K., Gomez J.P., Fromberg A., Granby K., Rosowski J., Castillo O., Pedreschi F. (2013). Are Chileans exposed to dietary furan?. Food Addit. Contam. Part A.

[36] Altaki M.S., Santos F.J., Puignou L., Galceran M.T. (2017). Furan in commercial baby foods from the Spanish market: Estimation of daily intake and risk assessment. Food Addit. Contam. Part A.

[37] Roberts D., Crews C., Grundy H., Mills C., Matthews W. (2008). Effect of consumer cooking on furan in convenience foods. Food Addit. Contam. Part A.

[38] Kim T.-K., Lee Y.-K., Park Y., Lee K.-G. (2009). Effect of cooking or handling conditions on the furan levels of processed foods. Food Addit. Contam. Part A.

[39] Sirot V., Rivière G., Leconte S., Vin K., Traore T., Jean J., Carne G., Gorecki S., Veyrand B., Marchand P. (2019). French infant total diet study: Dietary exposure to heat-induced compounds (acrylamide, furan and polycyclic aromatic hydrocarbons) and associated health risks. Food Chem. Toxicol..

[40] Cilla A., Alegria A., De Ancos B., Sanchez-Moreno C., Cano M.P., Plaza L., Clemente G., Lagarda M.J., Barberá R. (2012). Bioaccessibility of Tocopherols, Carotenoids, and Ascorbic Acid from Milk- and Soy-Based Fruit Beverages: Influence of Food Matrix and Processing. J. Agric. Food Chem..

[41] Cilla A., Bosch L., Barberá R., Alegría A. (2018). Effect of processing on the bioaccessibility of bioactive compounds–A review focusing on carotenoids, minerals, ascorbic acid, tocopherols and polyphenols. J. Food Compos. Anal..

[42] Aguilera J.M. (2005). Why food microstructure?. J. Food Eng..

[43] Zhou L.-Y., Li W., Pan W.-J., Sajid H., Wang Y., Guo W.-Q., Cai Z.-N., Wang D., Yang W.-W., Chen Y. (2017). Effects of thermal processing on nutritional characteristics and non-volatile flavor components from *Tricholoma lobayense*. Emir. J. Food Agric..

[44] Akıllıoğlu H.G., Bahçeci K.S., Gökmen V. (2015). Investigation and kinetic evaluation of furan formation in tomato paste and pulp during heating. Food Res. Int..

[45] Seok Y.-J., Her J.-Y., Kim Y.-G., Kim M.Y., Jeong S.Y., Kim M.K., Lee J.-Y., Kim C.-I., Yoon H.-J., Lee K.-G. (2015). Furan in Thermally Processed Foods-A Review. Toxicol. Res..

[46] Shen M., Zhang F., Hong T., Xie J., Wang Y., Nie S., Xie M. (2017). Comparative study of the effects of antioxidants on furan formation during thermal processing in model systems. LWT.

[47] Limacher A., Kerler J., Conde-Petit B., Blank I. (2007). Formation of furan and methylfuran from ascorbic acid in model systems and food. Food Addit. Contam..

[48] Mogol B.A., Gökmen V. (2013). Kinetics of Furan Formation from Ascorbic Acid during Heating under Reducing and Oxidizing Conditions. J. Agric. Food Chem..

[49] Owczarek-Fendor A., De Meulenaer B., Scholl G., Adams A., Van Lancker F., Yogendrarajah P., Uytterhoeven V., Eppe G., De Pauw E., Scippo M.-L. (2010). Importance of Fat Oxidation in Starch-Based Emulsions in the Generation of the Process Contaminant Furan. J. Agric. Food Chem..

[50] Adams A., Bouckaert C., Van Lancker F., De Meulenaer B., De Kimpe N. (2011). Amino Acid Catalysis of 2-Alkylfuran Formation from Lipid Oxidation-Derived α,β-Unsaturated Aldehydes. J. Agric. Food Chem..

[51] Märk J., Pollien P., Lindinger C., Blank I., Märk T. (2006). Quantitation of Furan and Methylfuran Formed in Different Precursor Systems by Proton Transfer Reaction Mass Spectrometry. J. Agric. Food Chem..

[52] Becalski A., Forsyth D., Casey V., Lau B.P.-Y., Pepper K., Seaman S. (2005). Development and validation of a headspace method for determination of furan in food. Food Addit. Contam..

[53] Shen M., Liu Q., Jiang Y., Nie S., Zhang Y., Xie J., Wang S., Zhu F., Xie M. (2015). Influences of Operating Parameters on the Formation of Furan During Heating Based on Models of Polyunsaturated Fatty Acids. J. Food Sci..

[54] Limacher A., Kerler J., Davidek T., Schmalzried F., Blank I. (2008). Formation of Furan and Methylfuran by Maillard-Type Reactions in Model Systems and Food. J. Agric. Food Chem..

[55] Locas C.P., Yaylayan V.A. (2004). Origin and Mechanistic Pathways of Formation of the Parent FuranA Food Toxicant. J. Agric. Food Chem..

[56] Van Lancker F., Adams A., Owczarek-Fendor A., De Meulenaer B., De Kimpe N. (2011). Mechanistic Insights into Furan Formation in Maillard Model Systems. J. Agric. Food Chem..

[57] Varelis P., Hucker B. (2011). Thermal decarboxylation of 2-furoic acid and its implication for the formation of furan in foods. Food Chem..

[58] Delatour T., Huertas-Pérez J.F., Dubois M., Theurillat X., Desmarchelier A., Ernest M., Stadler R.H. (2020). Thermal degradation of 2-furoic acid and furfuryl alcohol as pathways in the formation of furan and 2-methylfuran in food. Food Chem..

[59] Mariotti-Celis M.S., Zúñiga R., Cortés P., Pedreschi F. (2017). A Kinetic Study of Furan Formation in Wheat Flour-Based Model Systems during Frying. J. Food Sci..

[60] Palmers S., Grauwet T., Celus M., Wibowo S., Kebede B., Hendrickx M.E., Van Loey A. (2015). A kinetic study of furan formation during storage of shelf-stable fruit juices. J. Food Eng..

[61] Al-Baali A.G.A.-G., Farid M.M., Al-Baali A.G.A.-G., Farid M.M. (2006). Principles of Thermal Sterilization. Sterilization of Food in Retort Pouches.

[62] Hauck C., Ramirez C., Simpson R. (2017). Efecto del Procesamiento Térmico de Zanahoria (d. carota) en la Biodisponibilidad de ß-Caroteno Obtenida Mediante Estudios In Vitro.

[63] Erdoǧdu F., Balaban M.O. (2003). Nonlinear Constrained Optimization of Thermal Processing II. Variable Process Temperature Profiles to Reduce Process Time and to Improve nutrient Retention in Spherical and Finite Cylindrical Geometries. J. Food Process. Eng..

[64] Chen C., Ramaswamy H. (2004). Multiple Ramp-variable Retort Temperature Control for Optimal Thermal Processing. Food Bioprod. Process..

[65] Noronha J., Hendrickx M., Suys J., Tobback P. (1993). Optimization of Surface Quality Retention During the Thermal Processing of Conduction Heated Foods Using Variable Temperature Retort Profiles. J. Food Process. Preserv..

[66] Salgado C., Ramirez C., Simpson R., Nuñez H. (2019). Aplicación de Perfiles Variables de Temperatura (VRT) para la Esterilización Simultánea de Multiprocesos.

[67] Blakiestone B., Heldman D., Moraru C. (2010). Retortable Pouches. Encyclopedia of Agricultural, Food and Biological Engineering.

[68] Lebowitz S.F., Bhowmik S.R. (1990). Effect on Retortable Pouch Heat Transfer Coefficients of Different Thermal Processing Stages and Pouch Material. J. Food Sci..

[69] Shah M.A., Bosco S.J.D., Mir S.A., Sunooj K. (2017). Evaluation of shelf life of retort pouch packaged Rogan josh, a traditional meat curry of Kashmir, India. Food Packag. Shelf Life.

[70] Ghai G., Teixeira A.A., Welt B.A., Goodrich-Schneider R., Yang W., Almonacid S. (2011). Measuring and Predicting Head Space Pressure during Retorting of Thermally Processed Foods. J. Food Sci..

[71] Bindu J., Ravishankar C., Gopal T.S. (2007). Shelf life evaluation of a ready-to-eat black clam (*Villorita cyprinoides*) product in indigenous retort pouches. J. Food Eng..

[72] Tribuzi G., De Aragão G.M.F., Laurindo J.B. (2015). Processing of chopped mussel meat in retort pouch. Food Sci. Technol..

